# Circadian Clock Gene *Period* Contributes to Diapause via GABAeric-Diapause Hormone Pathway in *Bombyx mori*

**DOI:** 10.3390/biology10090842

**Published:** 2021-08-30

**Authors:** Wen-Zhao Cui, Jian-Feng Qiu, Tai-Ming Dai, Zhuo Chen, Jiang-Lan Li, Kai Liu, Yu-Jun Wang, Yang-Hu Sima, Shi-Qing Xu

**Affiliations:** 1 School of Biology and Basic Medical Sciences, Medical College, Soochow University, Suzhou 215123, China; 20184021015@stu.suda.edu.cn (W.-Z.C.); jfqiu@suda.edu.cn (J.-F.Q.); 20214021004@stu.suda.edu.cn (T.-M.D.); 1730402072@stu.suda.edu.cn (Z.C.); 20194221011@stu.suda.edu.cn (J.-L.L.); 20194221050@stu.suda.edu.cn (K.L.); simyh@suda.edu.cn (Y.-H.S.); 2 Institute of Agricultural Biotechnology & Ecology (IABE), Soochow University, Suzhou 215123, China; 3 Guangxi Key Laboratory of Beibu Gulf Marine Biodiversity Conservation, College of Marine Sciences, Beibu Gulf University, Qinzhou 535011, China; wanghong@bbgu.edu.cn

**Keywords:** *Bombyx mori*, circadian clock, *Period*-knockout mutant, diapause, *GRD*

## Abstract

**Simple Summary:**

Diapause in insects is a classic and long-term concern subject regulated by both circadian clock and endocrine system. Studies in many insects have shown that disturbance of circadian clock system can affect diapause occurrence. However, the specific molecular regulation mechanism and key nodes between circadian clock and endocrine hormones regulating diapause occurrence are still lack of insightful reports. Our work identified the molecular nodes and pathways through which the transcription-translation feedback loop of the silkworm circadian clock regulated the level and action of diapause hormones, based on the diapause change in a silkworm mutant line of *Period* gene knockout. This work confirmed that *Period* knocked out in silkworms changed the classic temperature- and photoperiodic-dependent diapause-destiny and changed the diapause through the GABA-DH neurotransmitter-endocrine hormone pathway, and showed that the GABA receptor gene, *GRD*, was controlled by both the circadian clock and endocrine system in silkworms. The results provided an example to explain the regulatory mechanism of the circadian clock on endocrine hormones in the silkworm.

**Abstract:**

Diapause is a developmental transition in insects based on seasonal adaptation to adversity; it is regulated by a circadian clock system and the endocrine system. However, the molecular node and its mechanism underlying the effects of these systems are still unclear. Here, a mutant of *Bombyx mori* with the circadian clock gene *Period* (*Per*) knocked out was constructed, which dramatically changed the classic diapause-destined pathway. *Per*-knockout silkworms powerfully attenuated, but could not completely block, the predetermined effects of temperature and photoperiod on diapause determination, and this effect depended on the diapause hormone (DH) pathway. The impaired transcription-translation feedback loop of the circadian clock system lacking the *Per* gene caused direct up-regulation of the expression of *GRD*, a receptor of γ-aminobutyric acid (GABA), by changing expression level of *Cycle*. The synthesis of GABA in the tissue complex of brain-suboesophageal ganglion then increased and restricted the decomposition, which continuously promoted the GABAergic signal to play a role, and finally inhibiting (delaying) the release of DH to the hemolymph, and reducing the diapause-inducing effect of DH. The results provided an example to explain the regulatory mechanism of the circadian clock on endocrine hormones in the silkworm.

## 1. Introduction

The coordinated regulation of the circadian clock and endocrine system on the physiology and behavior is a fascinating and confusing subject that has attracted much research attention. Studies have shown that the circadian clock regulated the organism’s metabolism and endocrine systems, so that the body could adapt to environmental temperature, light, and diet [1,2,3]. This is mediated by a series of transcription factors through the mutual regulation of the transcription-translation feedback loop (TTFL) and endocrine hormones [4,5,6]. However, many investigators believe that circadian rhythms and metabolic processes also have a reciprocal interaction [7,8,9].

Studies in animal models such as mice have found that the central circadian clock located in the hypothalamus can directly regulate the release of reproductive axis hormones [10]. Interfering with the *mClock* gene, a core member of the TTFL of the circadian clock of mammals, results in the suppression of estrogen synthesis in ovarian cells, while interfering with the *mPer2* gene increases the content of progesterone [11]. Knocking out the *mBmal1* gene resulted in decreased insulin secretion in mice [12]. Knockout of the *DrPer1b* gene in zebrafish (*Danio rerio*) resulted in an attention deficit and hyperactive behaviors caused by the decrease of dopamine levels in the brain [13]. Research on *Drosophila melanogaster* has found that the brain affects hormone secretion by regulating the peripheral circadian clock of endocrine organs [14]. Knockout of the *DmPer* circadian clock gene inhibited the synthesis of steroid hormones [14,15]. Studies on insect *Per* genes and physiological functions have found that disrupting the neuronal regions of the brain expressing the *PtPer* gene affected the behavioral rhythm and the ratio of diapause in *Protophormia terraenovae* [16]. The reproductive of *Riptortus pedestris* is induced by the suppression of juvenile hormone (JH) secretion [17]. Reducing the *RpPer* transcription level by RNAi changed the expression pattern of JH regulatory genes, leading to non-diapause under the conditions of diapause induction but this effect on diapause could be saved by supplementing JH analogues [18]. However, most existing reports have only emphasized correlations between the circadian rhythm and level of endocrine hormones and metabolites. The studies showed that the circadian clock had regulatory effects on the levels of endocrine hormones and metabolites, but the molecular regulation mechanism is still unclear.

The *Bombyx mori* (*B. mori*) silkworm is an important economic insect in animal husbandry, and the silk protein secreted by silkworms is a valuable raw material in the textile and biomedical engineering industries [19,20]. *B. mori* is the only model insect of Lepidoptera, which comprises up to 70% of agricultural and forestry pests [21,22]. Silkworm diapause is an ideal trait for studying the synergistic mechanism of temperature and light, the two main zeitgebers. The circadian clock signal is the initial signal pathway to control the silkworm diapause. Temperature induces activation of the silkworm diapause eggs, while it is the combined effect of temperature and light in the late stage of parental embryonic development that determines the diapause of silkworms, and clearly the effect of temperature is strongly greater than that of light [23,24,25,26]. A problem that has persisted in the fields of sericulture and entomology for nearly 100 years is that the mechanism of temperature- and light-inducing diapause has been associated with the role of the diapause hormone (DH) [27,28,29], and the molecular nodes determining how diapause is controlled by both the circadian clock and endocrine system have not been identified in silkworms. The focus of this study was therefore to identify the molecular nodes and pathways through which the TTFL of the silkworm circadian clock regulated the level and action of diapause hormones.

## 2. Materials and Methods

### 2.1. Silkworm Strain and Rearing

A bivoltine race of silkworms named DAZAO was used in the experiments, and its egg-diapause phenotype was determined by the mother’s experience of the temperature and photoperiod during embryonic development. A constant incubation temperature of 25 °C (25), 20 °C (20), or 15 °C (15), and one type of daily illumination of continuous light (LL), continuous darkness (DD), or 12 h light followed by 12 h darkness (LD), which were combined into six types of incubation conditions, that were 25LL, 25LD, 25DD, 20LL, 20DD, and 15LD. After hatching, the larvae were fed with fresh mulberry leaves at 25LD (±1.5 °C) and relative humidity 75–85% until adulthood. Figure S1 shows the effect of the environment experienced by maternal embryos on the diapause of next generation eggs. Pupation time of each larva was accurately recorded (±1 h). Hemolymph, ovary, and brain-suboesophageal ganglion complex (Br-SG) of female pupae were collected at pupal ages of 24 h, 48 h, 72 h, 96 h, and 120 h (±2 h), and the samples were stored at −80 °C.

The injection was performed on female pupae at the intersegment membrane of the third abdominal segment, with a glass capillary needle diameter ≤ 100 µm. The DH (sequence: TDMKDESDRGAHSERGALWFGPRL, purity ≥ 98%) was synthesized by Sangon Biotech (Shanghai, China), and picrotoxin was from Apexbio (B5054; Apexbio, Houston, TX, USA). Each pupa was injected with DH 10 µL (0.5 µg/µL or 1.0 µg/µL) or picrotoxin (30 µL of 3 µg/µL). The DH injection was performed at pupal age 72 h, and the picrotoxin injection was performed at 24 h, 48 h, 72 h, 96 h, and 120 h (±2 h). The negative control was injected with the same volume of sterile water.

The egg-diapause was investigated at 48 h after laying. Regarding the percentage of dark brown eggs peculiar to diapause eggs that appeared in the egg batch, ≥90% was recorded as the diapause egg batch (D), ≤10% as the non-diapause egg batch (ND), and 10–90% as the mixed egg batch (MD) of diapause and non-diapause.

### 2.2. Plasmid Construction and Per Gene Knockout Mutant Screening

**Figure 1:**
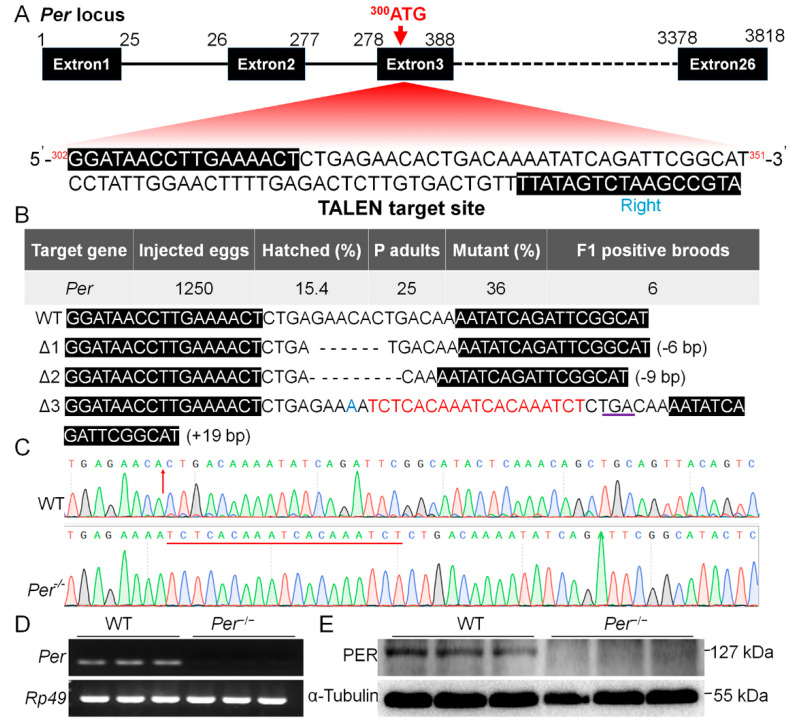
The target site for knockout *Period* gene (*Per*) and the mutant screening in *B. mori.* (**A**) Gene structure and the TALEN knockout sequences (302G...351T) designed for the transcription start site (300ATG) located on the third exon of *Per*. (**B**) Mutant screening efficiency and the 3 types of *Per* gene mutations screened. (**C**) Sequencing peak map of ∆Per-3 mutant. (**D**,**E**) Verification of *Per* knockout effect on mRNA level and protein level respectively. The material is the newly hatched larvae (*n* = 3 batches, 200–300 larvae per batch).

TALEN-mediated genome editing technology was used to knockout the silkworm *period* gene (*Per*). The knockout target was located at +19 bp from the transcription start site of exon 3, and the length was 16 bp (Figure 1A). The TALEN plasmids were digested with *Not I* (FD0594; Thermo Fisher Scientific, Waltham, MA, USA), the linearized plasmid was treated with proteinase K, and was purified by phenol/chloroform extraction (*v*:*v* = 1:1) and absolute ethanol precipitation.

The purified plasmid templates were then transcribed as cap-mRNAs using a SP6 transcription kit (AM1340; Invitrogen, Carlsbad, CA, USA), and further purified with LiCl to obtain cap-mRNA-L (450.6 ng/μL)/-R (460.8 ng/μL). The embryos (eggs) were incubated at 15LD, which induced (determined) the later adults to lay non-diapause eggs. The preparative cap-mRNA-L/R (*v*:*v* = 1:1) was injected into the non-diapause eggs within 8 h of egg age using a microinjector (IM300; Narishige, Setagaya-ku, Tokyo, Japan), with an injection amount of approximately 5–10 ng/egg. After injection, the eggs were incubated at 25LL, relative humidity 80–90% for hatching. G0 generation adults were mated with wild-type (WT) adults, and the mutants were screened by detecting the individual DNA sequences of the moths after oviposition. The PCR primers are shown in Table S1. From the G1 generation, the moths were brother and sister inbred, using continuing DNA sequencing until a homozygous mutation (records, *Per^−^*^/*−*^) was found in a batch.

### 2.3. Brain Transplantation

The female pupae with ages of 10 h (±1 h) were prefixed for 2 h at a low temperature of 4 °C. According to previous methods [30], a small incision was made in the ventral corneum of the head with microsurgical clips, then the complete brains of *Per*-knockout and wild-type were interchanged and transplanted (*n* = 15). The incision was sealed with nontoxic glue, and the postoperative pupae were kept in a sterile moisturizing petri dish until adults emerged. An intact ovary removed from every silkworm moth aged 3–6 h was used to assay the contents of 3-hydroxykynurenine, the serosal pigment precursor specific to diapause eggs, which was used to evaluate the diapause destiny of eggs.

### 2.4. Western Blot Analysis

The PER protein was analyzed by western blotting. The larvae (*n* ≥ 30) within 2 h after hatching were homogenized with cell lysis buffer (P0013; Beyotime, Shanghai, China), centrifuged (4 °C, 13,800× *g*, 20 min), and the protein concentration in the supernatant was assayed using the BCA method (pc0020; Solarbio, Beijing, China). The protein was resolved using SDS-PAGE. The gel was transferred to a polyvinylidene difluoride membrane for immunoblotting (1620177; Bio-Rad, Hercules, CA, USA), blocked in blocking buffer (P0023B; Beyotime), and then incubated with primary antibody. The membrane was then washed with TBST three times (5 min each wash) and incubated with the secondary antibody, followed by use of the ChemiDoc™ Imaging System (12003153; Bio-Rad). The PER protein (NCBI Reference Sequence: NP_001036975.1) has total lengths of 1108 amino acid residues, and the antigen of PER polyclonal antibody is length of 592 amino acid residues from M^1^ to T^592^. Rabbit primary antibody against PER was synthesized by Wuhan GeneCreate Biological Engineering (Wuhan, China) and the secondary antibody was goat anti-rabbit (GAR0072; Lianke Bio, Hangzhou, China). The antibody dilutions were 1:1000 and 1:5000 for primary and secondary antibody, respectively.

### 2.5. Quantitative Real-Time PCR

Total RNA was extracted from the newly hatched larva, ovaries of female pupae, and tissue complex of Br-SG using TRIzol reagent (15596018; Invitrogen) according to the manufacturer’s instructions. The RNA concentration was determined using a spectrophotometer (Nanodrop 2000; Thermo Fisher Scientific). A Prime Script^™^ RT reagent Kit (RR037B; TaKaRa, Dalian, China) was used for cDNA synthesis. Quantitative real-time PCR (qRT-PCR) was performed to measure gene mRNA levels using the TB Green^®^ Premix Ex Taq^™^ (RR420B; TaKaRa) in an ABI StepOnePlus^™^ Real-Time PCR system (Ambion, Foster City, CA, USA). Primer sequences were designed on the BLAST website (Table S1). The qRT-PCR reaction system was 20 μL, with reaction conditions of 95 °C for 30 s, followed by 40 cycles of 95 °C for 5 s and 60 °C for 34 s, with a final melting curve of 95 °C for 15 s, 60 °C for 60 s, and 95 °C for 15 s. The mRNA level for individual gene was normalized using the *Rp49* gene as a reference, and transcript levels were quantified using the 2^−^^ΔΔCT^ method [31,32].

### 2.6. RNA Interference

Two kinds of double-stranded RNA (dsRNA) were injected to interfere in the expression of the glutamic acid decarboxylase gene (*GAD*). The length of both dsRNAs was 21 bp, targeting 333G-351A (dsRNA-GAD-333) and 1691G-1709A (dsRNA-GAD-1691). A dsRNA mixture containing 5 µg each of dsRNA-GAD-333 and dsRNA-GAD-1691 was diluted in 10 µL ddH_2_O, then injected into a 48-h-old pupa (50–60 females). The negative control was injected with the same volume of ineffective (negative) interference dsRNA (NC). The injection and diapause judgment of laid eggs were conducted by the same method as the DH injection.

### 2.7. Liquid Chromatography-Mass Spectrometry/Mass Spectrometry (LC-MS/MS) Analysis

LC-MS/MS was used to determine the DH level in hemolymphs, and the contents of γ-aminobutyric acid (GABA) [33] and dopamine (DA) [34] in hemolymphs or Br-SG tissue complexes. A total of 150 µL of phosphate-buffered saline (PBS) was added to one Br-SG sample (from 50 female pupae), followed by homogenization (FastPrep-24; MP Biomedicals, Burlingame, CA, USA). The pretreatment of the Br-SG homogenate and hemolymph samples was as follows: 30 µL of sample was added to 90 µL of 0.1% formic acid/acetonitrile. After mixing, the supernatant was extracted by centrifugation (18,800× *g*, 6 min). DH, GABA (03835; Sigma-Aldrich, St. Louis, MO, USA), and dopamine (DA)(43658; Sigma-Aldrich) were determined by the full scanning electrospray ionization (+) mode (M/Z: 80–990). The quantitative ion was 547.5 > 159.2, 104.0 > 87.0, and 153.9 > 137.1 and the qualitative ion was 547.5 > 442.2, 104.0 > 69.0, and 153.9 > 119.1, respectively. An ACQUITY UPLC BEH HILIC column (1.7 µm, 2.1 × 100 mm; Waters, Milford, MA, USA) was used in the LC-MS/MS (5500; AB SCIEX, Redwood City, CA, USA) system at 40 °C with a sample volume of 5 µL. The mobile phase A was 0.1% FA, and B was acetonitrile. The initial mobile phase B was 20% acetonitrile for DH, and 98% acetonitrile for GABA and DA, and the flow rate was 0.3 mL/min for both.

### 2.8. Ehrlich’s Diazo Reaction

As previously reported [35,36], Ehrlich’s diazo reagent was used to detect 3-hydroxykynurenine [37]. On the day of adult emergence, complete ovaries (including eggs) were dissected out from the moth, washed with PBS, dried on filter paper, weighed, and homogenized with 3% Na_2_CO_3_ solution (*w*:*v* = 1:5). After centrifugation (9600× *g* for 5 min), the supernatant was collected as the sample solution. According to the sample solution, Ehrlich’s diazo reagent = 1:2 (*v*:*v*), the color was compared by being photographed or detected using the absorbance value at A490 nm within 5 min after mixing. 

### 2.9. Assay of Trehalose and Glycogen

The contents of trehalose and glycogen were measured using the anthrone reaction. The absorbance at 620 nm was measured using a microplate reader (Synergy HT; Biotek, Winooski, VT, USA). The standard products were trehalose (S11052; YuanYe, Shanghai, China) and glucose (G6172; Macklin, Shanghai, China). Trehalose assay steps involved adding 150 µL 80% ethanol to 10 µL of the hemolymph sample, and heating in water for 20 min at 75 °C. After centrifugation (1500× *g* for 10 min), the supernatant was resuspended and the pellet was washed with 80% ethanol, then combined with the two supernatants. After evaporating to dryness using a 65 °C water bath, 20 µL ultrapurified (UP) water was added to dissolve the sample. Then, 30 µL 6 M NaOH was added, and heated for 10 min to 100 °C to destroy reducing sugars. Finally, an anthrone reaction was performed. The glycogen assay steps involved weighing 0.1 g of the ovarian sample, adding 500 µL 30% KOH (*w*:*v*), and heating for 30 min at 100 °C. After cooling, 750 µL absolute ethanol was added, and the sample was then placed on ice for 10 min, followed by centrifugation (900× *g* for 10 min). The precipitate was resuspended successively with 500 µL UP water, 10 µL saturated KCl, and 1000 µL absolute ethanol, and incubated for 5 min at 60 °C. The precipitate from centrifugation (900× *g* for 10 min) was dissolved in 1000 µL UP water and used as the anthrone reaction sample.

### 2.10. Chromatin Immunoprecipitation and Sequencing (ChIP-Seq)

The BmN cells were collected in logarithmic growth phase according to the instructions of the Pierce Magnetic ChIP Kit (26157; Thermo Fisher Scientific), then formaldehyde corresponding to 1% of total volume was added, followed by crosslinking at room temperature for 10 min. Then, 1/10 volume of glycine solution (10×) was added at room temperature for 5 min to stop the crosslinking reaction. The cells were then washed twice with precooled PBS, and 10 μg of antibody was added for the immunoprecipitation. The rabbit anti-CYCLE and rabbit anti-CLOCK antibodies were produced by Wuhan GeneCreate Biological Engineering (Wuhan, China). The CYCLE and CLOCK proteins had total lengths of 700 and 647 amino acid residues, respectively. Their polyclonal lengths were 399 amino acids (170–568) and 308 amino acids (30–337), respectively. The DNAs collected by ChIP from the CYCLE and CLOCK reactions were sent to GENEWIZ (Suzhou, China) for library construction and sequencing. Bowtie2 software (https://www.dnv.com/services/bow-tie-software-for-analysis-and-risk-assessment-barrier-management-synergi-life-39311?utm_campaign=qhse_synergi_life&utm_source=google&utm_medium=cpc&gclid=Cj0KCQjw--GFBhDeARIsACH_kdZlk2mDZtUWjmeg6HnNjPcmPyRJCfXpNKu7W-rAfyWac4i9iwScLg8aAtNlEALw_wcB&gclsrc=aw.ds, accessed on 27 August 2021) was used to compare and annotate the silkworm reference genome database.

### 2.11. Dual Luciferase Reporter Assays

The 450 bp fragment of the *GRD* gene promoter region of the silkworm containing three E-boxes was amplified and cloned into the luciferase reporter gene vector, pGL4.10 (Promega, Madison, WI, USA), and was named *GRD luc*. The full-length cDNA of silkworm *Clock*, *Cycle*, and *Per* were cloned into the pcDNA3.1(+) expression vector. The mouse 293T cell line was used for transfection according to the manufacturer’s instructions for Lipofectamine™ 2000 Transfection Reagent (11668019; Thermo Fisher Scientific). After 24 h of transfection, the Dual-Luciferase^®^ Reporter Assay System (E1910; Promega) was used to detect luciferase activity using a fluorescence and chemiluminescence analyzer Fluoroskan Ascent™ FL (Thermo Fisher Scientific).

### 2.12. Statistical Analysis

Prism 8 (GraphPad, San Diego, CA, USA) was used for statistical calculations and graph construction. Significance analysis was performed using a *t*-test and data were expressed as the mean ± SEM. According to a previous report [38], JTK_CYCLE software was used to analyze the rhythm of circadian clock gene expressions within 24 h.

## 3. Results

### 3.1. Per Expression Is Necessary for Diapause-Destiny in Bombyx mori

The designed TALEN target (Figure 1A) was used to knock out the silkworm *Period* gene (*Per*). After microinjecting 1250 eggs, 193 larvae were obtained (hatching rate 15.4%). The genomic DNA of G0 generation adults were detected after mating and oviposition, and 9 mutants were detected from 25 adults (mutation rate 36%), with 6 G1 positive broods obtained (Figure 1B). Further screening showed three types of mutations in the heterozygous state of the G1 generation, namely ∆*Per*-1 with a deletion of 6 bp, ∆*Per*-2 with a deletion of 9 bp, and ∆*Per*-3 with a replacement of 1 bp and an insertion of 19 bp (Figure 1B). 

We selected a 19 bp insertion and formed a stop codon (∆*Per*-3), resulting in a protein truncation by more than 99% for homozygous screening. Using continuous sequencing selection of self-generations, a homozygous mutant was obtained in the G5 generation (Figure 1C). Identification of gene transcription and translation levels showed that *Per* mRNA and protein could not be detected in this mutant line (Figure 1D,E). *Per* gene was stably knocked out with continuous 12 generations of genome testing, which was denoted as *Per*^−/−^ in this study.

Next, we investigated transcriptional changes of the core member genes of the TTFL of the circadian clock. The results showed that the transcription levels of *Cry1*, *Cry2*, *Tim*, *Clock*, and *Cycle* genes differed, when compared with the WT, in which the expression of *Cry1* and *Clock* in *Per*^−/−^ silkworms lost circadian rhythms, indicating that knocking out the *Per* gene disrupted the transcriptional rhythm patterns of core members of the TTFL of the silkworm circadian clock (Figure S2). This may have further affected the signal output of the TTFL of the circadian clock system. 

**Figure 2:**
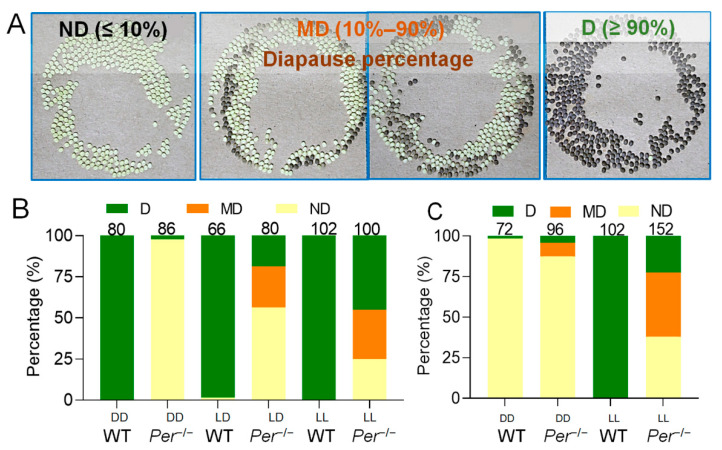
*Per*-knockout affects the determinative effect of incubation temperature and light on the diapause of offspring eggs. (**A**) Three diapause phenotypes of eggs (batches) produced by female moths. ND, non-diapause egg batch, the proportion of diapause eggs ≤ 10%; MD, the mixed batch of diapause eggs and non-diapause eggs, the proportion of diapause eggs was 10–90%; D, the diapause egg batch, the proportion of diapause eggs was ≥90%. In the photos, the yellow eggs are non-diapause eggs, and the dark brown eggs are diapause eggs. (**B**) At 25 °C incubation temperature, the effect of the incubation light regimes (DD, LD and LL) on the diapause of offspring eggs (*n* = 66–102 batches). (**C**) At 20 °C incubation temperature, the effect of the incubation light regimes (DD and LL) on the diapause of offspring eggs (*n* = 72–152 batches). The numbers of the egg batches are marked above the corresponding column.

Investigating the phenotype of the mutant silkworms showed that a non-diapause change of eggs occurred (Figure 2). The phenotypic change was stable when investigated for multiple generations. Incubation of embryos in an invariable 25LD environment resulted in almost all of the female moths of the WT group laying diapause eggs (D). In the *Per* knockout group, nearly 60% of female moths laid non-diapause eggs (ND), aproximately 25% of the moths laid mixed diapause and non-diapause eggs (MD), and less than 20% moths laid diapause eggs (D) (Figure 2A,B). Further investigations the effect of the incubation environment of 25DD or 25LL on the diapause of offspring eggs, the results showed that almost all postembryonic female moths of the WT group laid D-type eggs, while that of the diapause-type of the *Per* knockout group was powerfully affected by incubation light in the parental embryonic-period. The D-type of the *Per*^−/−^ offspring eggs was less than 3.0% in 25DD, and increased to 45.0% in 25LL. The percentage of D-type batches instigated by 25LD was higher than that of 25DD, but lower than that of 25LL (Figure 2B). Under incubation temperature of 25 °C, the day length did not affect the diapause decision in WT, and the female moths laid diapause eggs. But the day length played a decisive role for the *Per* knockout silkworms, and the diapause rate increased as extension of day length (25LL > 25LD > 25DD) with a dose effect.

When the parent eggs were incubated at an intermediate temperature of 20 °C, the light cycle of incubation had a decisive effect on the diapause of offspring eggs of the WT group, but it had little effect on that of the *Per* knockout group. The female moths of the D-type egg-laying moths in the WT group increased from 3.0% for the 20DD to 100% for the 20LL, while it increased from 12.5% at the 20DD to 22.5% at the 20LL in the *Per* knockout group (Figure 2C). It is worth noting that the effect of light on the diapause of the *Per* knockout group at 20 °C was lower than that at 25 °C (Figure 2B,C). In conclusion, knocking out the *Per* gene resulted in weakening of the temperature and light sensitivities of the silkworm embryos, which were decisive for diapause.

### 3.2. Deletion of Per Gene Inhibited the Secretion of DH through GABA Pathway in Brain

**Figure 3:**
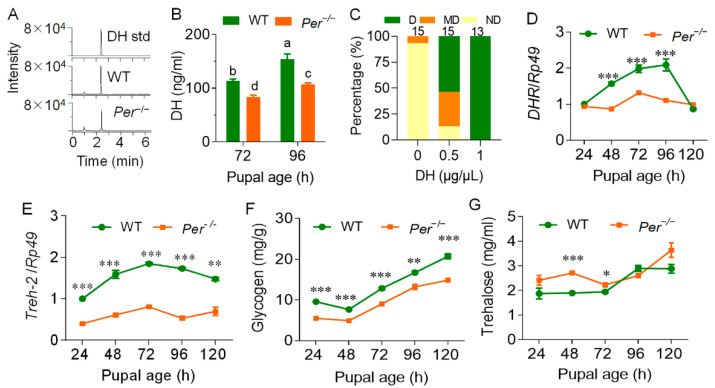
*Per*-knockout affects the DH content and the diapause metabolism of the female pupal hemolymph. The parental silkworm eggs were incubated at 25LD, and the larval and pupal stage maintained a 25LD environment. (**A**) DH mass spectrum. DH std, standard product of DH; WT and *Per*^−/−^ represents the 72 h pupal hemolymph of wild-type and mutant silkworm, respectively. (**B**) DH content in pupal hemolymph. In the multiple comparison of significant differences in the figure, there is no significant difference between the same letters, and there are significant differences between different letters (*p* ≤ 0.05, *n* = 3). (**C**) The diapause rescue effect of DH supplementation by female pupae on the eggs laid by mutant adults (*n* = 13–15 batches). The parental eggs were incubated at 25LD, and the pupa was injected 10 µL DH at age 72 h (±2 h). DH concentration (*w*:*v*) was 0.5 µg/µL and 1.0 µg/µL. The control was injected with the same volume of sterile water. The transcription levels of *DHR* (**D**) and *Treh-2* (**E**) in the pupal ovaries were measured by qRT-PCR and with the reference gene was *Rp49* (*n* = 3). (**F**) Glycogen content in the ovary (*n* = 4). (**G**) The content of trehalose in hemolymph (*n* = 6). In Figure (**B**–**G**), data were expressed as the mean ± SEM, the difference between *Per*^−/−^ and WT is: *, *p <* 0.05; **, *p <* 0.01; ***, *p <* 0.001.

To identify the role of DH in the non-diapause phenotype of *Per* mutant silkworms, the level of DH during the critical period when Br-SG released DH to the hemolymph was determined. LC-MS/MS results showed that from 72 h to 96 h of pupal age, DH levels in the WT female pupal hemolymph were significantly increased, indicating that Br-SG positively affected the release of DH to hemolymphs. Although the DH level in the *Per* knockout group also increased, it was obviously lower than that in the WT group during the same period (Figure 3A,B).

To characterize the rescue effect of DH, which induces the occurrence of silkworm eggs diapause on *Per* mutant silkworms, the female pupae were injected with synthetic DH at a pupal age of 72 h. When supplementing with 5 µg DH per pupa, the percentage of *Per*^−/−^ diapause moths (D-type) increased from 0.0% to 53.4%. In addition, the percentage of non-diapause moths decreased from 93.3% in the negative control to 13.3%. When each pupa was supplemented with 10 µg of DH, 100% of the eggs laid by the female moths were diapause egg batches (Figure 3C). These results showed that DH induced an efficient rescue effect on *Per* knockout silkworm diapause, and also indicated that knocking out the *Per* gene may have reduced the levels and effect of DH in pupal hemolymphs, leading to non-diapause.

The mRNA levels of the DH-encoding gene, diapause hormone (DH)-pheromone biosynthesis activating neuropeptide (PBAN) *DH-PBAN*, and its transcription factor gene, *Pitx*, were measured during the pupal age from 24 h to 120 h. The results showed that in the Br-SG tissues that synthesized and secreted DH, there was no significant difference between these two gene transcription levels between the *Per* mutant and the WT group (Figure S3). We further determined the transcription level of the DH receptor gene, *DHR*, in the ovary, and found that during the pupal age of 48–96 h, the critical period of DH secretion in the WT group, the expression of this gene in the *Per*^−/−^ silkworms was severely downregulated, when compared with the WT group. However, after 96 h, there was no significant difference between the groups (Figure 3D).

The transcription levels of diapause metabolism markers and rate-limiting enzyme genes triggered by the diapause hormone were then measured. The *trehalase-2* gene (*Treh-2*) transcription level in the mutant ovary was significantly downregulated, when compared with the WT group at the pupal ages of 24 h–120 h (Figure 3E). The glycogen content in the ovary was always lower than that of the WT group (Figure 3F), while the trehalose level in the hemolymph had a tendency to be higher than that of WT group before the pupal age of 72 h, with no significant difference thereafter (Figure 3G). Together, the results showed that the weakened DH signal of the mutant *Per^−/−^* silkworms affected the conversion and transport of trehalose in hemolymphs.

Although the previous results showed that the mutant silkworms with knockout of the *Per* gene had decreased DH levels in the pupal hemolymph, and the response of ovarian cells to the DH receptor was weakened, the weakened response did not result from the downregulation of gene transcription of the *DH* gene. Using brain transplantation experiments, these results further proved the relationship between the DH activity levels and its effects on changes of the brain in *Per* knockout silkworms. 

**Figure 4:**
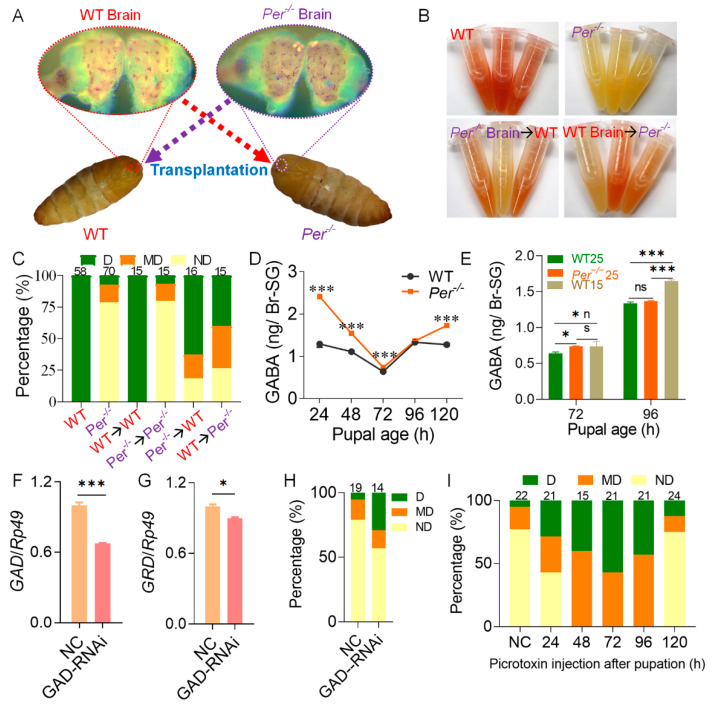
Deletion of *Per* affects diapause via the neurotransmitter GABA in the pupal brain. (**A**) Schematic of brain transplantation. Brain transplantation was performed on female pupae with ages of 10 h (±1 h). (**B**) Effect of brain transplantation on the content of 3-hydroxykynurenine. The 3-hydroxykynurenine content of intact ovaries from moth ages of 3–6 h was detected using Ehrlich’s diazo reaction. (**C**) Effect of brain transplantation on egg diapause. Absorbance of Ehrlich’s diazo reaction was used to judge the diapause types according to the diapause classification criteria (*n* = 15–70 female moths). (**D**,**E**) The content of GABA in Br-SG of pupae. The parental embryonic incubation was 25LD (**D**), 25LD and 15LD (**E**). (**F**,**G**) Efficiency of interference with *GAD* on the diapause of *Per*^−/−^. GAD-RNAi, *GAD* interference dsRNA was injected at pupal age of 48 h. NC, invalid interference fragments were injected. The mRNA levels of *GAD* (**F**) and *GRD* (**G**) in Br-SG were measured 24 h after injection (*n* = 3). (**H**) Effect of GAD-RNAi on diapause. The ND batches rate was 79% in NC and the ND rate declined to 57% after GAD-RNAi (*n* = 14–19 batches). (**I**) Picrotoxin treatment can rescue the diapause of *Per^−/−^*. 90 μg picrotoxin was injected into each *Per^−/−^* pupa. The control (NC) was injected with 30 μL sterile water at pupal age of 24 h. The diapause was determined by the content of 3-hydroxykynurenine in the moth ovaries ages of 3–6 h (*n* = 15–24 female moths). In figure (**D**–**G**), * *p* < 0.05; *** *p* < 0.001, *n* = 3. In figures (**F**–**I**), eggs were incubated in 25LD.

At the pupal age of 10 h (±1 h), we interchanged and transplanted *Per*^−/−^ and WT female pupal brains that were not connected to SG lost the characteristic of carrying DH (Figure 4A). The adults rarely laid eggs after emergence, and the diapause could not be judged by the color of the eggs. Because the specific serosal molecular pigment precursor of diapause eggs is 3-hydroxykynurenine in ovaries, the contents in virgin moths (moth age of 3–6 h) were detected using the Ehrlich’s diazo reaction. According to the diapause classification criteria of the color reaction (Figure S4), the content of 3-hydroxykynurenine increased in the ovaries of *Per^−/−^* moths implanted with WT brains, and the diapause rate increased. On the contrary, the content of 3-hydroxykynurenine in the ovaries of WT moths implanted with *Per^−/−^* brains decreased, and the diapause rate decreased (Figure 4B,C). It indicated that deletion of *Per* reduced the diapause of offspring eggs by affecting the control of brain on DH secretion. This supported the result that the DH signal was weakened in *Per*^−/−^ middle and late pupae. Because the influence of the donor-carrying DH or the potential synthesis of donor SGs and secretion of DH was excluded during brain transplantation, the results described above indicated that the donor brain affected DH synthesis and secretion of SG.

We further determined the content of the neurotransmitters, DA and GABA, which regulate DH secretion after stimulation and inhibition in the brain. The LC-MS/MS results showed that during the pupal age of 24–120 h of the WT group, the content of DA in the Br-SG complex was rapidly reduced from the high level of 24 h, and was at a very low level for 48 h. In the Br-SG of mutant *Per*^−/−^ pupae, the DA content at the pupal age of 24 h had lower levels than that of the WT group, with a low level of oscillation during the period of 48–120 h (Figure S5).

We then determined the content of GABA, which inhibited DH secretion in the Br-SG. When the embryonic stage silkworms were exposed to the 25LD diapause-predetermined environment, the GABA content of the *Per* knockout group was higher than that of WT silkworms. Except for the pupal age of 96 h, which was not statistically significant, the other pupal ages all reached the *p* < 0.001 level; but, during the pupal age of 24–96 h, the GABA level gradually approached that of WT silkworms (Figure 4D). Further comparisons with the WT silkworm in 15LD non-diapause incubation environment (lay non-diapause eggs) showed that the GABA-content of the 25LD-*Per*-knockout group increased to the level of the 15LD-WT group at a pupal age of 72 h, while it restored to the lower level of the previous 25LD-WT group at a pupal stage of 96 h (Figure 4E). Our data indicated that the DH secretion period was delayed in the Br-SG of the *Per* knockout pupae.

At 24 h after the dsRNA interference of *GAD*, the mRNA levels of *GAD* and the GABA receptor gene, *GRD* in the Br-SG were then determined. The mRNA level of *GAD* was downregulated by approximately 33% in Br-SG (Figure 4F), and the expression of *GRD* was also downregulated by about 10% (Figure 4G). The percentage of diapause egg batches (D + MD) increased from 21% in the NC group to 43% (Figure 4H).

Further treatment of the *Per* knockout pupae with the GABA receptor blocker, picrotoxin, showed that reducing the effect of GABA rescued the diapause process of the *Per*^−/−^ silkworms. After injection with picrotoxin during pupal age 24–120 h, according to the diapause classification criteria of Ehrlich’s diazo reaction, the evaluation results showed that when picrotoxin was injected at 48 h, 72 h, or 96 h of pupal age, the percentage of diapause-destined moths (D + MD) was 100%. In addition, the effect of picrotoxin injection at the pupal age of 24 h was also better than that of the NC, and the percentage of D-type plus MD-type moths in the group was nearly 2-fold higher than that in the NC. However, the injection of picrotoxin at the pupal age of 120 h had almost no effect on the promotion of diapause (Figure 4I). The results indicated that the decrease of DH level in pupal stage of the *Per* gene knockout mutant caused the production of non-diapause eggs, which was closely related to the stronger role of the GABA neurotransmitter in Br-SG, that inhibited DH secretion during the specific period of the pupal ages of 48 h–96 h.

### 3.3. Per Directly Inhibited GABA Receptor GRD through Circadian Activators CYCLE and CLOCK

**Figure 5:**
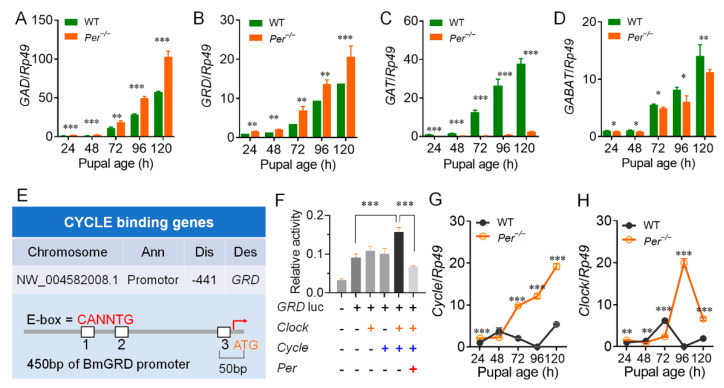
*Per^−/−^* affects the transcriptional regulation of key genes in the GABA pathway. (**A**–**D**) Gene transcription levels in the GABA pathway. *GAD*, glutamic acid decarboxylase; *GRD*, ionotropic GABA receptor; *GAT*, plasma membrane GABA transporter; *GABAT*, GABA transaminase. (**E**) ChIP-seq identification of CYCLE binding genes. Ann, Annotation; Dis, Distance to TSS; Des, Description. Genome alignment revealed that the promoter region of GRD has a binding site for CYCLE. (**F**) Dual luciferase reporter assays. *GRD* is activated by CLOCK and CYCLE, and inhibited by PER (sample replicates, *n* = 4). (**G**,**H**) The transcription levels of *Cycle* and *Clock*. In figures (**A**–**D**) and (**G**,**H**), eggs were incubated in 25LD. qRT-PCR was used to determine the genes transcription levels in Br-SG (*n* = 3). *, *p* < 0.05, **, *p* < 0.01, ***, *p* < 0.001.

We next investigated the transcription levels of GABA pathway-related genes in the Br-SG tissue complex. Within 24–120 h of pupal age, the transcription level of the GABA synthase gene, *GAD* (Figure 5A), and the GABA receptor gene, *GRD* (Figure 5B), were upregulated in the *Per* knockout silkworm compared with the WT, although the other four GABA receptor-subunit genes (*RDL1*, *RDL2*, *RDL3*, and *LCCH3*) showed inconsistent up- and down-regulated expressions (Figure S6A–D). Further studies showed that the plasma membrane GABA transporter gene, *GAT* (Figure 5C), and the GABA transaminase gene, *GABAT* (Figure 5D), which controlled the decomposition process of GABA, had significantly downregulated mRNA levels, while the transcription level of the *GAT* gene was downregulated many times in the *Per* knockout silkworms. However, the vesicle transporter gene of GABA, *VGAT*, the succinate semialdehyde dehydrogenase gene (*Ssadh*), and the degradation enzyme of the primary breakdown product of GABA knockouts had almost the same transcription levels as WT silkworms (Figure S6). These results showed that the decomposition of GABA in the Br-SG tissue complex of the *Per* knockout silkworms was limited, while the synthesis and effects of GABA were enhanced.

To further explain the molecular mechanism of the non-diapause effects in the silkworms caused by the knockout of the *Per* gene, we screened CYCLE and CLOCK, two circadian clock transcription regulators, for their regulatory effects on members of the DH pathway. ChIP-seq was used to screen the genes regulated by CYCLE/CLOCK in silkworm BmN cells. Then, the promoter sequence of the *GRD* gene was bound by the CYCLE protein. The E-box was located −441 bp from the transcription start site (Figure 5E). Luciferase assays further showed that co-transfection of the *Cycle* and *Clock* genes of the silkworm obviously increased the activity of the *GRD* gene promoter, but when co-transfected with the *Per* gene of the infected silkworm, it severely reduced the activity of the *GRD* promoter (Figure 5F). These results showed that transcription of silkworm *GRD* was regulated by the transcriptional regulatory factors of the circadian clock system.

We investigated the mRNA levels of core clock genes in the Br-SG tissue complexes, and found that within 24–120 h of pupal age, in contrast to the relatively stable transcription levels in the WT silkworm, the *Per* knockout silkworms continued to upregulate the expression of the *Cycle* gene, especially in the pupal ages of 72–96 h of the sensitive period of Br-SG release of DH (Figure 5G). In addition, the *Per* knockout silkworms continued to upregulate the expression of the *Clock* gene (Figure 5H) in the pupal ages of 48 h–96 h; the *Tim* and *Cry1/2* genes, which are core members of TTFL were also upregulated (Figure S7). Together, these results supported circadian clock transcription regulators of CYCLE/CLOCK upregulated the transcription level of the *GRD* gene.

## 4. Discussion

Among a variety of insects with adult reproductive diapause, the Hemipteran *Riptortus pedestris* [18,39,40], Diptera *Drosophila melanogaster* [41], and *Culex pipiens* [42] showed changes in reproductive diapause after knocking down or knocking out their *Per* genes. In the Hymenoptera pearl wasp (*Nasonia vitripennis*), the larval diapause was involved with the *NvPer* gene in the pupal stage, and the adult could not produce diapause-destined eggs even when exposed to short light conditions [43]. These results showed that the circadian clock system was involved in the regulation of insect diapause, but the molecular regulation mechanism is still unclear. An important reason is that the key model insect, *D. melanogaster,* lacks a noticeable diapause [44], which limits the progress of research on the endocrine and molecular genetics basis of the regulation of diapause by the circadian clock [45,46].

In the present study, using the classic egg-diapause model insect, *B. mori*, we found that knocking out the circadian clock gene, *Per*, changed the effects of temperature and light on the diapause determination of parent embryo stage. The mutant showed clear response to photoperiod on diapause at 25 °C, which was not observed in WT. In 25DD, exclusion of day length influence, the diapause rate of the mutant was remarkably lower than that of WT, and showed that knockout of *Per* gene weakened the decisive effect of 25 °C on diapause. When embryos were incubated at 25LD and 25LL, although day length had a compensatory effect on diapause determination, the weakening of temperature effect still existed. When embryos were incubated under 20 °C, the day length affected the diapause decision both in WT and mutant, the diapause rate increased as extension of day length (20LL > 20DD). The day length played a decisive role in diapause of WT, but the effect on mutants was dramatically weakened. These results showed that the circadian clock system damages in the *Per* knockout severely weakened, but could not completely block the decisive role of temperature and light during embryonic stages on the diapause of silkworm eggs.

(1) *Knockout of the circadian clock gene Per strongly changed the secretion and function of DH, which determined the diapause of silkworms.*

Diapause of insects including silkworms is an active adaptation to the adverse environment, but the occurrence of diapause of silkworm eggs does not depend on the temperature and photoperiod during the period of adverse environment, but the incubation environment of maternal embryos [23,47,48,49]. The results of the classic bivoltine silkworm showed that egg diapause is determined by the seasonal signal temperature, and day length experienced by the parent embryo during the later stages, which is reflected by the release of DH in the early and middle pupal stages. DH released into the hemolymph further affected diapause metabolism occurring in the middle and late pupal stages, then induced mature eggs to complete the diapause phenotype by the parent [50,51,52,53]. The environmental temperature and light in the later stages of the silkworm embryo, which determine diapause of the next generation of eggs, are essentially the zeitgeber of the circadian clock [54,55,56]. A basic scientific question is how the silkworm converts the timing signals of environmental temperature and light, which are sensed by the circadian clock, into storage signals to determine the endocrine level and role of diapause hormones in the pupal stage, thereby inducing subsequent diapause metabolism and the diapause phenotype of progeny eggs.

DH is synthesized in the pupal SG of silkworms, and is released into the hemolymph via the corpus cardiacum-corpus allatum complex in the 3–4 day pupal stage [57,58]. It then binds to the DH receptor (a G protein-coupled receptor) on the ovarian membrane [28,59], and activates trehalase in ovarian cells and the oocytes in the ovary [60,61], resulting in the trehalose in the hemolymph efficiently being converted into glucose and then being absorbed into the ovary, thereby promoting the accumulation of glycogen in the developing egg [29,62]. This is a prerequisite for the start of diapause metabolism of the silkworm. Notably, the activation of diapause eggs does not depend on the disappearance of DH, and even the presence of DH can be detected in non-diapause eggs [63].

In the present study, a mutant silkworm with knockout of the circadian clock, *Per* gene, showed a significant decrease in the DH content in the hemolymph at the pupal age of 72–96 h, which is the most sensitive time to induce diapause metabolism. The transcription levels of the DH receptor gene and the *trehalase-2* gene, two symbolic genes of the DH signaling pathway, were severely reduced in the ovary. The content of glycogen, a marker metabolite of diapause metabolism, showed typical non-diapause changes, whereas, supplementation with DH at an early pupal age completely repaired the non-diapause effect of *Per* knockout. These results showed that knocking out the *Per* circadian clock gene led to non-diapause in silkworm eggs, by reducing the level and effects of DH during the pupal stage.

(2) *The circadian clock of B. mori affected the DH levels and functions in the pupal stage through the GABA signaling pathway.*

Studies have shown that the DH response of silkworms has a strong sensitivity, depending on the developmental period. Only when the mother has a high DH level in the hemolymph of the pupal—age of 3–4 day, can the ovaries (including eggs) and other tissues be activated for diapause metabolism [50,57]. *DH-PBAN* is the DH-coding gene of the silkworm, which is specifically expressed in a spatiotemporal manner in the SG of the pupal stage, and is promoted by the transcription factor, Pitx [64,65]. The precursor protein expressed by the *DH-PBAN* gene can be sheared and processed into a variety of FXPRL amide neuropeptides, including DH, PBAN and three other SG nerve peptides (α-, β-, and γ-SGNPs) [66]. In the present study, although it was confirmed that non-diapause in the silkworm eggs resulted from knocking out the *Per* gene, which was caused by low DH levels in the pupal stage, the transcription level of the *DH-PBAN* gene and *Pitx* in the pupal stage Br-SG tissue complex were not downregulated. It is speculated that the post-translational shearing regulation of DH may not precisely regulate the DH levels of *Per*^−/−^ silkworms, and the regulation was more likely due to the secretion link of DH after shearing. Several studies have shown that non-diapause silkworm can synthesize the same amount of DH as diapause silkworm at pupal stage, but the secretion of DH is promoted and inhibited by the brain, resulting in different phenotypes of diapause of final offspring eggs, that is, the difference in DH secretion rather than synthesis ultimately affects the occurrence of diapause [23,57]. Moreover, three experiments, including brain transplantation, picrotoxin injection and *GAD* interference, all showed that promoting DH secretion could enhance the diapause effect of the mutant, indicating that the difference of DH titers was mainly the secretion regulation mechanism rather than the post transcriptional regulation mode.

The secretion of DH in the pupal stage of the silkworm is controlled by the brain [67,68]. The neurotransmitters, dopamine [69] and GABA [30,36,70] in the brain are responsible for the secretion of DH, and their roles are promoted and inhibited, respectively. GABA is synthesized by glutamic acid decarboxylase (GAD) [71], a major inhibitory neurotransmitter in the central system in insects [72,73,74] and vertebrates [33,75], and plays a role through GABAergic receptors in the optic tectum [76]. Silkworm GABA receptor subunit genes include *GRD*, *RDL1/2/3*, and *LCCH3* [77]. The Shiomi laboratory of Shinshu University (Nagano-ken, Japan) recently discovered that the expression of the silkworm GABA transporter (GAT) on the plasma membrane of the brain was temperature-dependent and modulated the DH release through the fine tuning of *GAT* gene expression levels. Embryos experiencing a low temperature of 15 °C had significant downregulation of *GAT* expression in the post-embryonic pupal stage, which further caused GABAergic signals to act on corazonin neurons for a long time, and ultimately this inhibited the release of DH [70].

In the present study, the brain interchange study results confirmed that the non-diapause phenotype of *Per* knockout mutant silkworms was caused by decreases in secretion of DH regulated by the brain. In tissues of the Br-SG, the GABA synthase *GAD* gene transcription level in *Per* knockout pupae was upregulated, when compared with WT pupae, and the GABA content increased to the level of non-diapause-destined pupa (15LD-WT) at 72 h, but decreased to the level of diapause-destined pupa (25LD-WT) at 96 h. The expression of the transaminase gene, *GABAT*, which catabolizes GABA, was also downregulated, and it was downregulated more significantly after 72 h of pupal age, suggesting that GABA break down in Br-SG was also reduced. In contrast, the expression of the GABA transporter gene, *GAT*, was dramatically downregulated compared with the WT, indicating that GABAergic signaling continued to be promoted in the pupal stage. Furthermore, interference of the GABA synthesis rate-limiting enzyme gene, *GAD*, in the pupal stage prevented the non-diapause changes of *Per* knockout silkworms. These results showed that the *Per* knockout mutant relied on the dual regulation of increased GABA synthesis and restricted breakdown, and this enhanced the inhibitory effect on DH secretion.

It was found in mice that GABA was the main neurotransmitter in the pacemaker cells, which was released in the suprachiasmatic nucleus according to a diurnal rhythm [75,78]. There were rhythmic level changes in different brain regions [79], suggesting that GABA was related to the circadian clock system. Though it has been proved that many regulators could affect the transcription of GABA receptor subunit genes, including cAMP response element-binding protein and methyl CpG-binding protein 2 [80,81,82,83]. Our study first report that the circadian clock directly regulates the expression of GABA receptor genes, *GRD* in silkworm. The present study found that CYCLE, a transcriptional regulator of the TTFL, bound to the −441 bp promoter region of the transcription start site of the *GRD* coding DNA sequence to directly regulate the transcription of the *GRD* gene of the GABA receptor. During the sensitive period of diapause hormone secretion from 72 h to 96 h of pupal age, the *Per* knockout silkworms also obviously upregulated expressions of *Cycle* and *Clock* genes in Br-SG, when compared with that of WT silkworms, and also upregulated the mRNA levels of the *GAD* and *GRD* genes. Further injection of the GABA receptor antagonist, picrotoxin, in the pupal stage showed that inhibition of the GABA receptor helped recovery of the diapause of the mutant. It showed that knocking out the silkworm *Per* gene affected the feedback regulation of the GABA pathway by changing the signal output of the circadian clock pathway.

## 5. Conclusions

**Figure 6:**
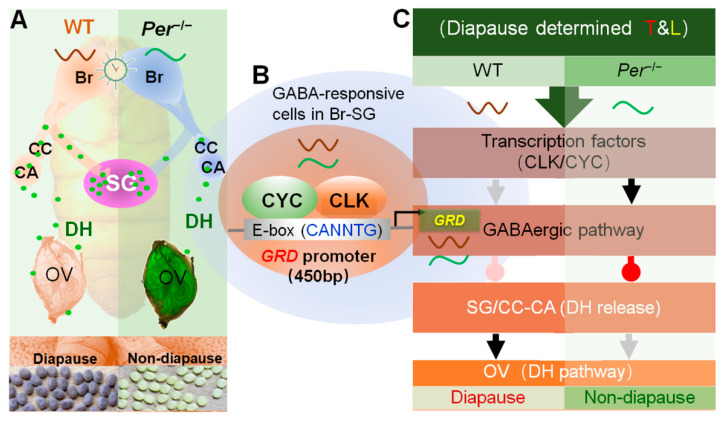
Summary about the diapause-destined mechanism changes in a circadian clock gene *Period* knockout mutant silkworm. Annotation: *Per*^−/−^, a *Per*-knockout strain of silkworm; Br-SG, brain-subesophageal ganglion complex; CC, corpus cardiacum; CA, corpus allatum; OV, ovary; T&L, temperature and light. (**A**) Knockout of *Per* changed the DH release in pupal stage. The pupal brain of *Per*^−/−^ weakened the regulation effect on DH secretion, resulted in the decrease of DH level in the hemolymph and delay of the titer peak, and finally resulted in non-diapause eggs laid by the female moths. (**B**) The GABAergic pathway is transcriptional regulated by a circadian clock transcription factor. The CLK/CYC, a complex of TTFL transcription factor of the silkworm, combined with the promoter region of the GABA receptor *GRD*, directly regulated the gene transcription level. (**C**) The circadian clock regulates the secretion and action of DH through the GABAergic pathway. In the pupal Br-SG, the CLK/CYC directly regulated the transcription of *GRD*, affected the GABAergic pathway, and finally inhibited (delayed) the release of DH to hemolymph, and reduced the diapause-inducing effects of DH. The gray and black arrows indicate weak and strong excitatory signal, and the pink and red water drop symbols indicate weak and strong inhibitory signal, respectively.

In conclusion, the diapause-destined mechanism changed in the circadian clock gene *Period* knockout silkworms (Figure 6). The circadian clock system directly upregulated the expression of *GRD* in the pupal stage through CYCLE, the transcriptional regulator, and feedback stimulated the increase in levels of the inhibitory neurotransmitter, GABA, thus continuing to promote the GABAergic signal in Br-SG, to inhibit the release of DH in hemolymphs.

## Data Availability

Not applicable.

## Supplementary Materials

The following are available online at https://www.mdpi.com/article/10.3390/biology10090842/s1, Figure S1: Effect of incubation environment of maternal embryos on diapause of eggs of the next generation, Figure S2: Deletion of *Per* affected the transcription of core member genes of TTFL in silkworm ovary at pupa stage, Figure S3: Transcription levels of DH related genes, Figure S4: Diapause classification criteria of the 3-hydroxykynurenine color reaction, Figure S5: Influence of Per knockout on the content of DA in Br-SG of pupae, Figure S6: Effects of *Per* gene knockout on the expression of GABAergic neurotransmitter related genes during the pupa stage, Figure S7: Effect of knocking out *Per* gene on the expression of clock genes in Br-SG of female pupae, Table S1: Sequence of primers and dsRNA.
