# Supplementary material for: Circadian Clock Gene Period Contributes to Diapause via GABAeric-Diapause Hormone Pathway in Bombyx mori
Source: Biology (Basel). 2021 Aug 30;10(9):842. doi: 10.3390/biology10090842 (PMC8469157; doi:10.3390/biology10090842)
Supplement: Supplementary file 1 [file biology-10-00842-s001.zip › biology-1327441-supplementary.pdf]

Supplementary Information for

Circadian clock gene *Period* contributes to diapause via GABAeric-diapause hormone pathway in *Bombyx mori*

Supplementary Figures

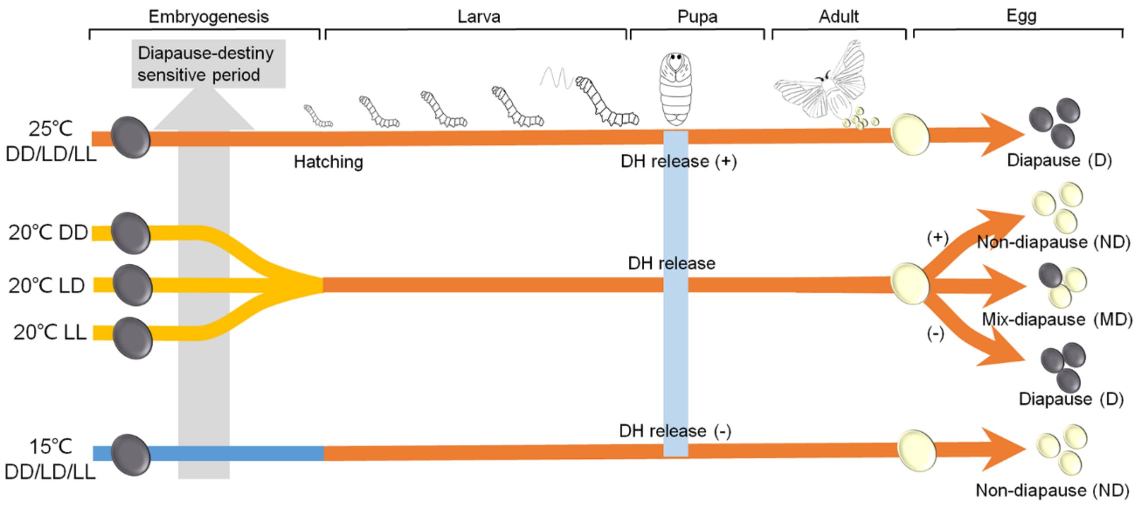

Supplementary Figure S1. Effect of incubation environment of maternal embryos on diapause of eggs of the next generation. The diapause of eggs is determined by the mother experienced the environment during their embryonic stage. The female moths lay diapause eggs if experienced a high temperature of 25 °C during their embryonic stage. The female moths lay non-diapause eggs if experienced a low temperature 15 °C during their embryonic stage. If the female moths experienced a intermediate temperature of 20 °C during their embryonic stage, the diapause of their spawn would be determined by day length.

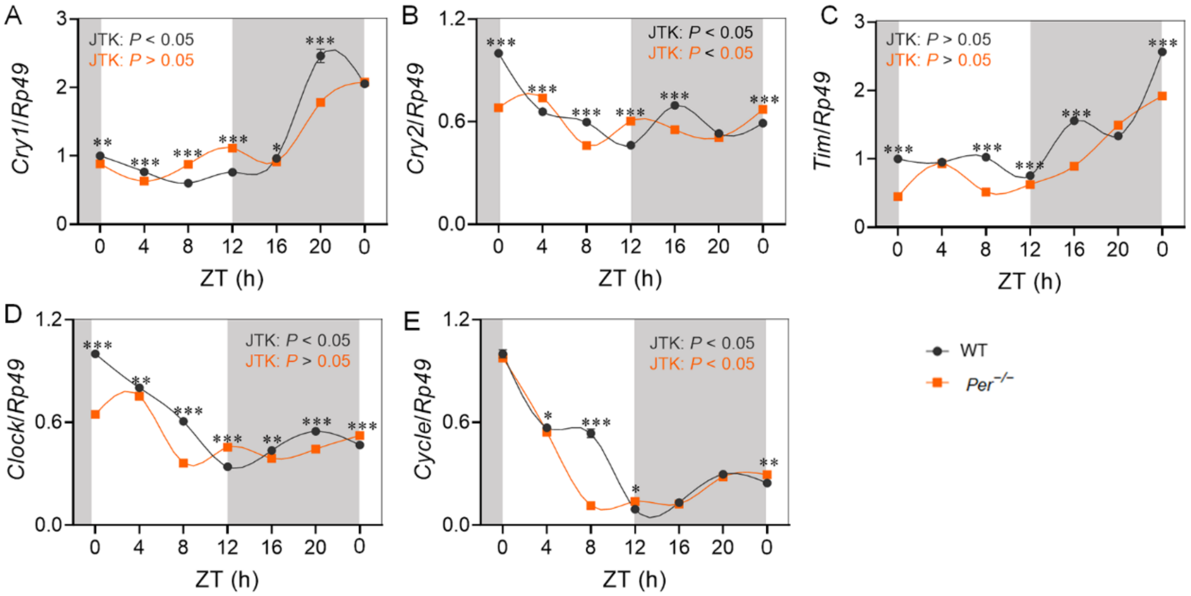

Supplementary Figure S2. Deletion of *Per* affected the transcription of core member genes of TTFL in silkworm ovary at pupa stage. ZT0 was set to pupal age 48 h (± 2 h), and RNA was extracted from ovaries every 4 h. The mRNA levels of *Cry1* (**A**), *Cry2* (**B**), *Tim* (**C**), *Clock* (**D**) and *Cycle* (**E**) were detected by qRT-PCR, and the reference gene was *Rp49*. The rhythm of gene transcription level in day and night (24 h) was analyzed by JTK_CYCLE software. JTK: $P > 0.05$ and $P < 0.05$ indicated that gene expression rhythm existed or not, respectively. The white and dark background represent light and dark periods, respectively. *, ** and *** indicates $P < 0.05$, $P < 0.01$ and $P < 0.001$ between $Per^{-/-}$ and WT, respectively (n = 3).

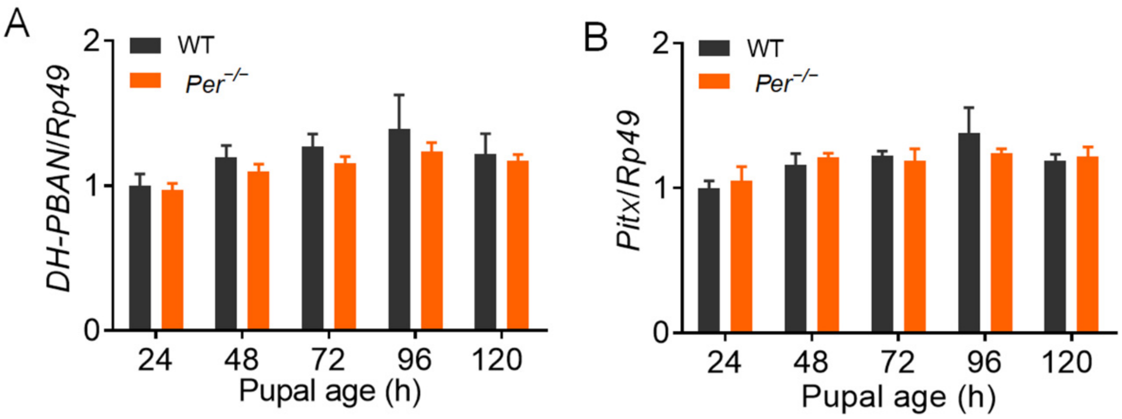

Supplementary Figure S3. Transcription levels of DH related genes. qRT-PCR was used to detect the transcription levels of DH synthesis gene *DH-PBAN* (**A**) and transcription factor *Pitx* (**B**) in Br-SG of female pupae. The reference gene was *Rp49*. $P > 0.05$, there was no significant difference between WT and *Per*−/− at all pupal ages (n = 3).

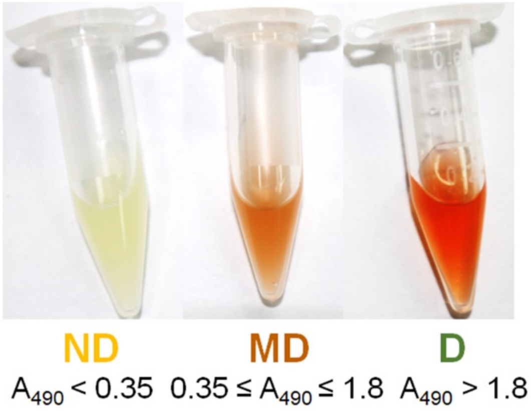

Supplementary Figure S4. Diapause classification criteria of the 3-hydroxykynurenine color reaction. Take the ND, MD and D-type batches as reference, the absorbance value of Ehrlich's diazo reaction was used as diapause classification criteria to evaluate the diapause of offspring eggs. A490 nm < 0.35 determined by Ehrlich's diazo reaction was defined as a non-diapause batch (ND), 0.35 ≤ A490 nm ≤ 1.8 was defined as a mixed batch (MD), A490 nm > 1.8 was defined as a diapause batch (D).

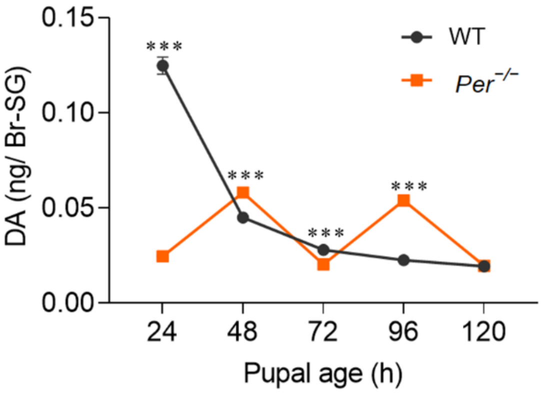

Supplementary Figure S5. Influence of *Per* knockout on the content of DA in Br-SG of pupae. LC-MS/MS were used to measure the content of DA in female Br-SG. The whole generation of silkworms were incubated at 25LD. The significance of difference is: *** $P < 0.001$. n = 3.

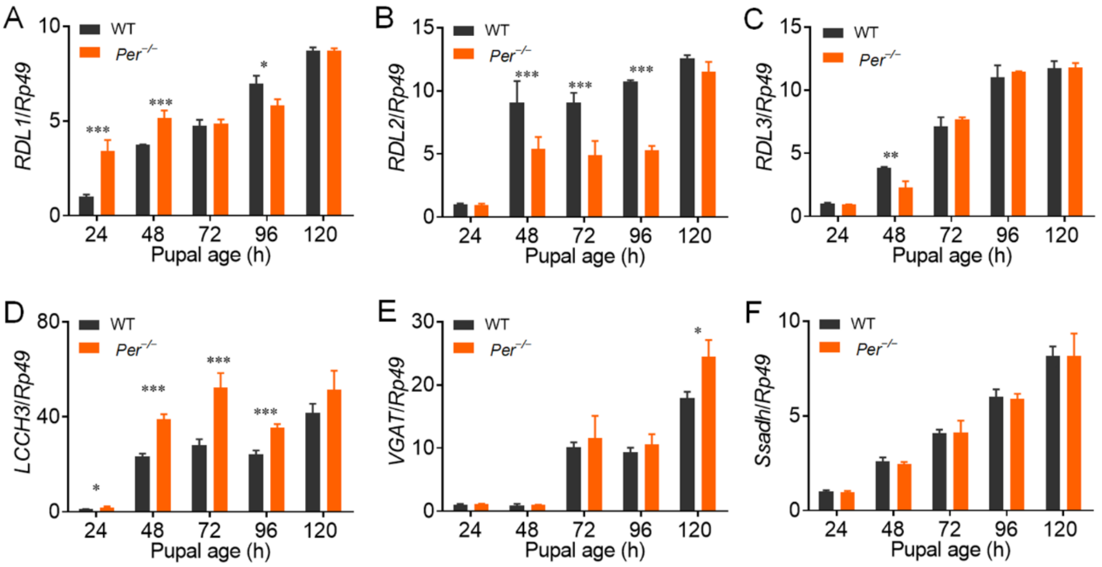

Supplementary Figure S6. Effects of *Per* gene knockout on the expression of GABAergic neurotransmitter related genes during the pupa stage. *RDL1*, *RDL2*, *RDL3* and *LCCH3* are the four receptor subunits of GABA; *VGAT*, vesicular GABA transporter; *Ssadh*, succinic semialdehyde dehydrogenase. After 25LD incubation for the eggs hatching, the larvae and pupae were under 25LD. qRT-PCR was used to determine the gene transcription levels of Br-SG in the female at pupal ages of 24-120 h (n = 3). The reference gene was *Rp49*. The significant difference is: * $P < 0.05$, ** $P < 0.01$, and *** $P < 0.001$.

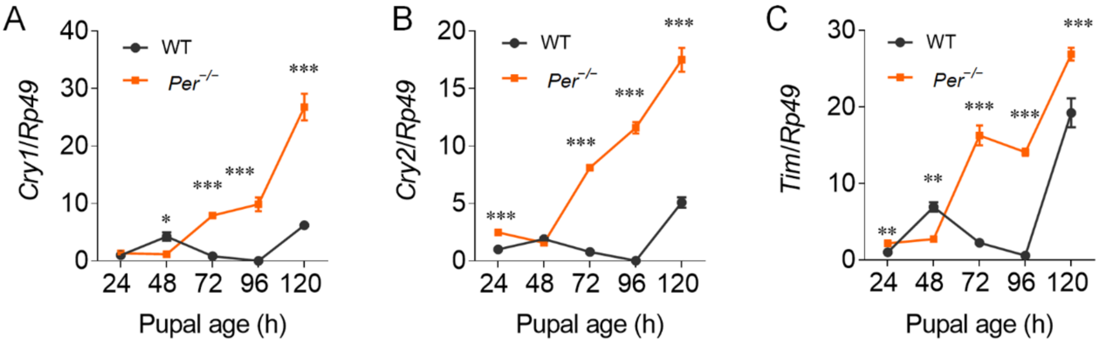

Supplementary Figure S7. Effect of knocking out *Per* gene on the expression of clock genes in Br-SG of female pupae. The eggs incubation, larval and pupal stages was maintained under 25LD. Gene transcription levels in the female Br-SG at pupal ages of 24 h to 120 h were measured by qRT-PCR. The reference gene was *Rp49*. The significance of the difference indicates that * $P < 0.05$, ** $P < 0.01$ and *** $P < 0.001$, n = 3.

Supplementary Table S1. Sequence of primers and dsRNA

Using	Gene name	Name	Sequence (5′-3′)
Genomic PCR	*Period*	Per-F	TTGGAAAAGTTTGTGGCTAATA
		Per-R	ACGATTGGCGATAGGGAAA
RT-PCR	*Period*	Per-F	GAAACGGAAACTGTATCGC
		Per-R	GAGGCAACAGAAGTAGTCA
	Rp49	Rp49-F	ACTCTGATGCTGAGCTGCTG
		Rp49-R	GACCTGTTTACAGGCCGACA
qRT-PCR	*Cry1*	Cry1-F	CCACGACAATCCGTCTCTT
		Cry1-R	GGTTGTACCCGACCACTTTCG
	Cry2	Cry2-F	TTGTCCGTGAAGAGAGAGCG
		Cry2-R	AGAGAGAAAAGCCTGGGTGG
	Tim	Tim-F	CTCTGCTCGGTCTTGTCATT
		Tim-R	TGCACGGCTTGAGACCATTA
	Clock	Clock-F	TGAACTCACATCCTGCTACT
		Clock-R	CTTTCTTGCTTGGCGTTT
	Cycle	Cycle-F	AAACGGAAACCATCGTCCTA
		Cycle-R	TTTGTTTCTTGTCGGGAGTG
	Treh-2	Treh-2-F	GTGTCGTTGCTGATCGTAGCA
		Treh-2-R	GCCCGTGGCAGTAAATCATAC
	DH-PBAN	DH-PBAN-F	AGCGATCAATGAAGCCATCCACTG
		DH-PBAN-R	TGCCTCTCGTAAGGTAGCTGGTC
	DHR	DHR-F	GACACCGCAAATGCTTCG
		DHR-R	ACCCAATAACCCTGATACAAATA
	Pitx	Pitx-F	GGTGTTGCTCCGTGCCCTTAC
		Pitx-R	TGATGAGTGCTGCTTCGCCTTC
	GAD	GAD-F	TACCAAGTCAAAACCGGGCA
		GAD-R	ATCAAGATGAAGACCGGGGC
	GRD	GRD-F	TTATCCTTGGCAGCTACGCC
		GRD-R	CGGTGTGCCTTTTGAGGTTG
	RDL1	RDL1-F	TCCAGAATGCCCTCCAG
		RDL1-R	AAAACGGACTTCAGATGGTCT
	RDL2	RDL2-F	TACCACCTAGCCGATCTTCG
		RDL2-R	TTGTCCTCCTGCTTCTTCGT
	RDL3	RDL3-F	TTCGCTACAAGGTCCGAGAT
		RDL3-R	GTTCATCCTGCTCCTGCTG
	LCCH3	LCCH3-F	ATCCACTCGACAGCCAGAAC
		LCCH3-R	TGGGGTAGTTCAGCGTCTTC
	Ssadh	Ssadh-F	TGAATTTGGCATGGTTGCTA
		Ssadh-R	CCTTCACGCCCTATACCAGA
	GABAT	GABAT-F	CAAGACCGGAAAGGTTTTGA
		GABAT-R	TCGGCCACGAACACTATGTA
	VGAT	VGAT-F	GGATGGAATTGCTCCAAGAA
		VGAT-R	TTTTAGCTCCTGGCTTTCCA
	GAT	GAT-F	TCTTCAATCTCGTGCAGTGG
		GAT-R	GTGACACGCCACAGGTACAC
	Rp49	Rp49-F	GCATCAATCGGATCGCTATG
		Rp49-R	GGACCTTACGGAATCCATTTG
RNAi	*GAD*	GAD-333-F	GCAAAUAGCAGAGCAUAUATT
		GAD-333-R	UAUAUGCUCUGCUAUUUGCTT
		GAD-1691-F	GGAUUCCUCAUGAUGCCAATT
		GAD-1691-R	UUGGCAUCAUGAGGAAUCCTT
